# Construction of miRNA–mRNA networks for the identification of lung cancer biomarkers in liquid biopsies

**DOI:** 10.1007/s12094-022-02969-7

**Published:** 2022-10-13

**Authors:** Elena Espinosa Garcia, Macarena Arroyo Varela, Rafael Larrosa Jimenez, Josefa Gomez-Maldonado, Manuel Angel Cobo Dols, M. Gonzalo Claros, Rocio Bautista Moreno

**Affiliations:** 1 Department of Computer Architecture, Universidad de Málaga, Malaga, Spain; 2 U.G.C. Medico-Quirurgica de Enfermedades Respiratorias, Hospital Regional Universitario de Málaga, Malaga, Spain; 3 Sequencing and Genomics Unit at SCBI, Universidad de Málaga, Malaga, Spain; 4 Area of Oncology and Rare Diseases (IBIMA), Hospital Regional Universitario de Málaga, Malaga, Spain; 5 Department of Molecular Biology and Biochemistry, Universidad de Málaga, Malaga, Spain; 6 Andalusian Platform for Bioinformatics at SCBI, Universidad de Málaga, Malaga, Spain; 7 Institute for Mediterranean and Subtropical Horticulture “La Mayora”, Universidad de Málaga and CSIC, Malaga, Spain

**Keywords:** High-throughput sequencing, RNA-seq, Biomarker, miRNA, Lung cancer

## Abstract

Lung cancer (LC) is the most common cause of cancer death worldwide mostly due to the low survival rate: 75% of cases are identified in advanced stages. In this study, the list of useful biomarkers to make an early diagnosis using liquid biopsies was expanded. A total of 30 samples of LC were analyzed to define potential miRNA biomarkers in liquid biopsies for LC. The biomarkers have been identified in interaction networks miRNA–mRNA. The potential biomarkers have been then validated in large cohorts. A total of 15 candidate miRNAs, that regulate the repression of 30 mRNAs, have been identified as a specific functional interaction network for squamous carcinoma, while the specific functional interaction network of adenocarcinoma consists of four candidate miRNAs that seem to handle the repression of five mRNA. Inspection of expression levels in larger cohorts validates the usefulness of the 11 candidates as biomarkers in liquid biopsies. The 11 candidate miRNAs found could be utilized to form diagnostic predictive biomarkers for LC in liquid biopsies.

**Supplementary Information:**

The online version contains supplementary material available at 10.1007/s12094-022-02969-7.

## Introduction

Lung cancer (LC) is the most common cause of cancer death worldwide; 1.6 million people die from this disease each year. It is estimated that in 2035, there will be 3 million annual deaths [1]. LC is categorized into two main histological groups: small cell lung carcinoma (SCLC) and non-SCLC (NSCLC), accounting for 15% and 85% of all lung cancers, respectively. NSCLCs are usually subcategorized into lung adenocarcinoma (LUAD), lung squamous cell carcinoma (lSCC), and large cell lung carcinoma (LCLC). It can be then deduced that LC is a heterogeneous disease, so accurate diagnosis is vital to provide the most appropriate treatment. In this sense, important advances in the treatment of LC have been achieved in the past decade. However, the survival rates for LC remain yet low since 75% of cases are identified in advanced stages. Since an early diagnosis through biomarkers is key to survival [2], new ways are needed to identify clinical biomarkers that could accelerate more accurate diagnosis, prognosis, and monitoring the evolution of the disease.

Spurred on by these challenges, studies based on the different types of RNA molecules, such as mRNA, sncRNA (miRNA and piRNA) and lncRNA, are becoming a profitable strategy for identifying biomarkers [3–5]. These molecules include miRNAs, small non-coding RNAs between 21 and 23 nucleotides in length, which have been shown to participate in gene regulation [6]. miRNAs can regulate transcription factors, tumor suppressor genes, and other regulatory elements that promote or inhibit cancer proliferation [7]. Moreover, miRNA–gene interactions have been reported to play an essential role in carcinogenesis. For example, miR-21, cluster miR-17-92 and miR-221/222 have been shown to act as oncogenes in lung tumor progression, while let-7 families, miR-34 and miR-200 act predominantly as tumor suppressors [8, 9]. In addition, some critical miRNAs for the diagnosis and treatment of cancer have recently been identified, coming from the analysis of circulating tumor cells, circulating miRNAs or miRNAs encapsulated in secreted microvesicles, such as exosomes [10–12]. A recent work implied that the breast cancer exosomes carrying miR-21 were more strongly associated with bone metastasis than with non-metastatic cancer or other metastatic sites  [13].

So, the main purpose of the present study is to expand the list of biomarkers useful to diagnose LC in liquid biopsies, based on the miRNA–mRNA interaction between tumor and healthy tissue. The candidate miRNAs have been validated in larger cohorts and liquid biopsy databases, confirming their diagnostic value. Moreover, the candidates hsa-miR-130-3p, hsa-miR-182-5p and hsa-miR-203-3p appear to come from exosomes secreted by tumor cells.

## Patients and methods

### Patient cohorts

Tumor and healthy tissue samples from seven patients with LUAD and 8 with lSCC, who have undergone surgical resection at the Hospital Regional Universitario de Málaga (HRUM), were selected from the Biobank of the Andalusian Public Health System (SSPA), as described in Arroyo et al. [14]. All samples have been recruited with the informed consent of the patients and have been authorized by the Malaga Provincial Ethics Committee. The patients were in tumor stages 1B–3A of the TNM classification of lung cancer, with only one in stage 1B, four in 2A, three in 2B, and seven in 3A, and had not undergone chemotherapy or radiotherapy treatments. The total RNA from 30 samples was extracted according to the biobank specifications as published in Arroyo et al. [14, 15]. The massive sequencing of the miRNAs was carried out in the Illumina®NextSeq550 sequencer of the Center for Supercomputing and Bioinnovation of the University of Malaga as described in Arroyo et al. [14]. Raw data generated for each of the samples are listed in Supplementary Table S1 (Table S1). Sequencing data are accessible on the BioProject PRJNA727087.

### Bioinformatics

**Fig. 1:**
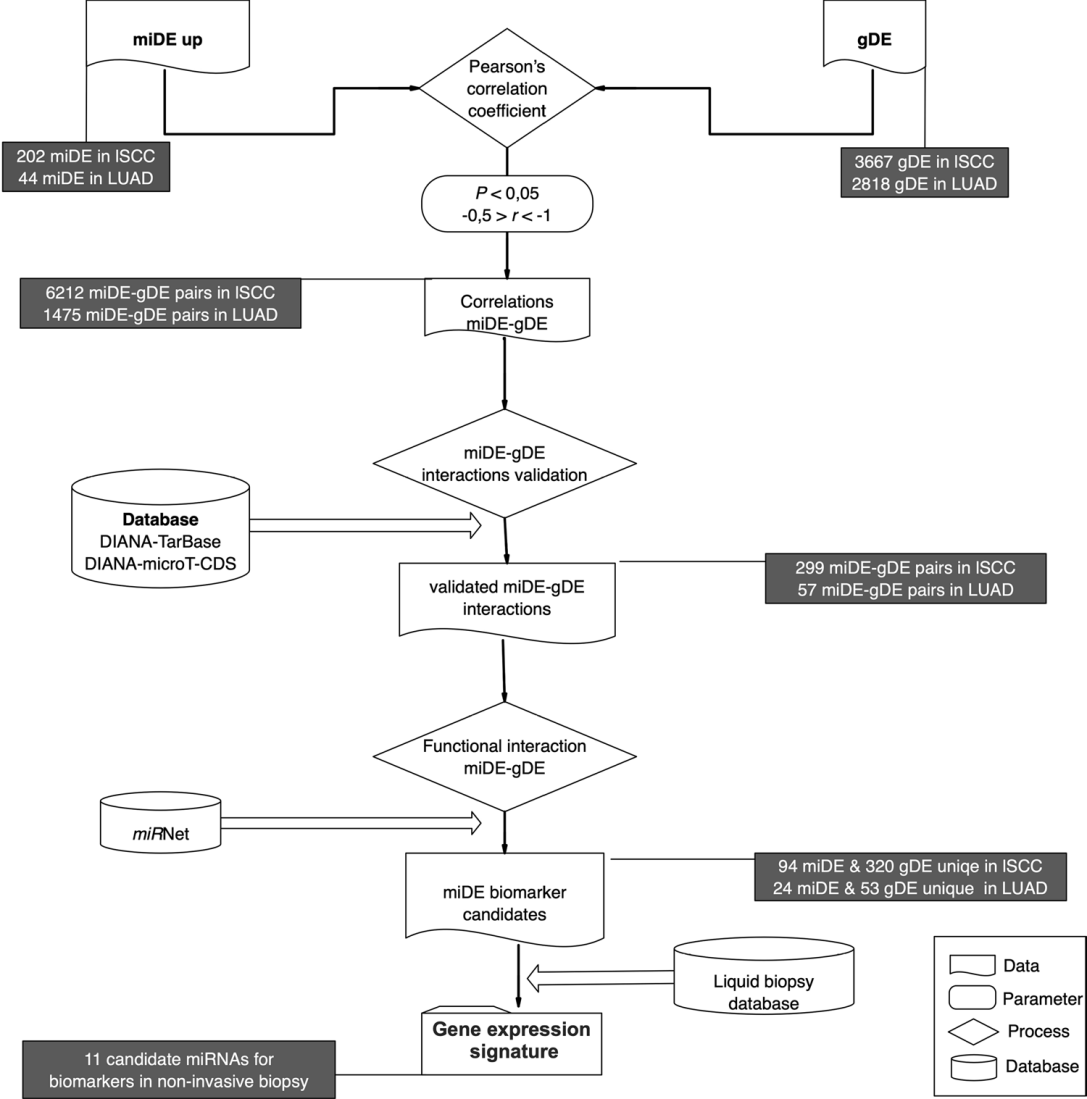
Workflow for the identification of biomarker candidate miRNAs

The analysis of miRNAs, their correlation with cellular mRNAs in the same samples, the study of miRNA–mRNA interaction and the verification of their possible biomarker nature are outlined in Fig. 1. More details about stages are given below.

#### Processing and differential expression

The raw data resulting from the miRNA sequencing were pre-processed with *SeqTrimNext* [16], eliminating low-quality reads, sequencing adapters, and any other type of contaminant. The useful reads were processed with *Oasis 2.0* [17], a pipeline that aligns on the hg38.p1 version (GCA_000001405.16) of the human genome and on the miRBase database v22.1 (http://www.mirbase.org/) using bowtie V.1 [18]. For each patient, miRNA expression levels were obtained as mapped reads per million (RPM). The calculation of the differential expression of miRNAs performed by *Oasis 2.0* is based on the R package *DESEq2* [19], using the default parameters. Differentially expressed miRNA (miDE) were those with an adjusted probability of $P < 0.05$ and a fold change (FC) of $|FC| > 2$. The set of differentially expressed genes (gDE) of these samples comes from a previously published work [14] in which we had identified 3667 gDE in lSCC and 2818 gDE in LUAD.

#### Correlation miDE–gDE

A script in *R* and *Python* (https://github.com/Unit-Bioinformatic-SCBI-UMA/miRNA-mRNA-correlation) has been developed to evaluate the Pearson’s correlation coefficient (*r*) between the FC values of the miDE and the gDE, for each patient in both types of LC. The significant miDE–gDE pairs, as defined by $-1< r < -0.5$ ($P < 0.05$), were selected. Significant miDE–gDE pairs were validated against databases of miRNA–mRNA interactions such as *DIANA-TarBase* [20] for the interactions validated experimentally and *DIANA-microT-CDS* [21] for predictively validated interactions. Finally, a specific interaction network was built, both for lSCC and LUAD, using *miRNet* [22] (https://www.mirnet.ca/). The functionality of the mRNAs in the network interaction has been studied by consulting *DAVID* [23].

A script in *R* and *Python* (https://github.com/Unit-Bioinformatic-SCBI-UMA/miRNA-mRNA-correlation) has been developed to evaluate the Pearson’s correlation coefficient (*r*) between the FC values of the miDE and the gDE, for each patient in both types of LC. The significant miDE–gDE pairs, as defined by $r < -0.5$ ($P < 0.05$), were selected. Significant miDE–gDE pairs were validated against databases of miRNA–mRNA interactions such as *DIANA-TarBase* [20] for the interactions validated experimentally and *DIANA-microT-CDS* [21] for predictively validated interactions. Finally, a specific interaction network was built, both for lSCC and LUAD, using *miRNet* [22] (https://www.mirnet.ca/). The functionality of the mRNAs in the network interaction has been studied by consulting *DAVID* [23].

#### Validation in large cohorts and liquid biopsies

The validation, in larger cohorts, of the expression changes of the candidate miDEs in the interaction network was carried out using *MIR-TV* [24] (http://mirtv.ibms.sinica.edu.tw/index.php) database, which stores 567 LUAD cases and 523 lSCC cases. Candidate miDEs were also inspected with the liquid biopsy database *liqDB* [25] (https://bioinfo5.ugr.es/liqdb/) to verify its presence in the extracellular fluids of plasma, serum and exosomes, and in the fluid of the bronchoalveolar lavage. Finally, to elucidate its possible secretion, *ERV* [26] (http://bioinfo.life.hust.edu.cn/EVmiRNA/) database describing the miRNA content of 461 extracellular vesicle samples, of which 34 are from lung cancer, was queried.

## Results

### Correlation miDE–gDE in LUAD and lSCC

**Fig. 2:**
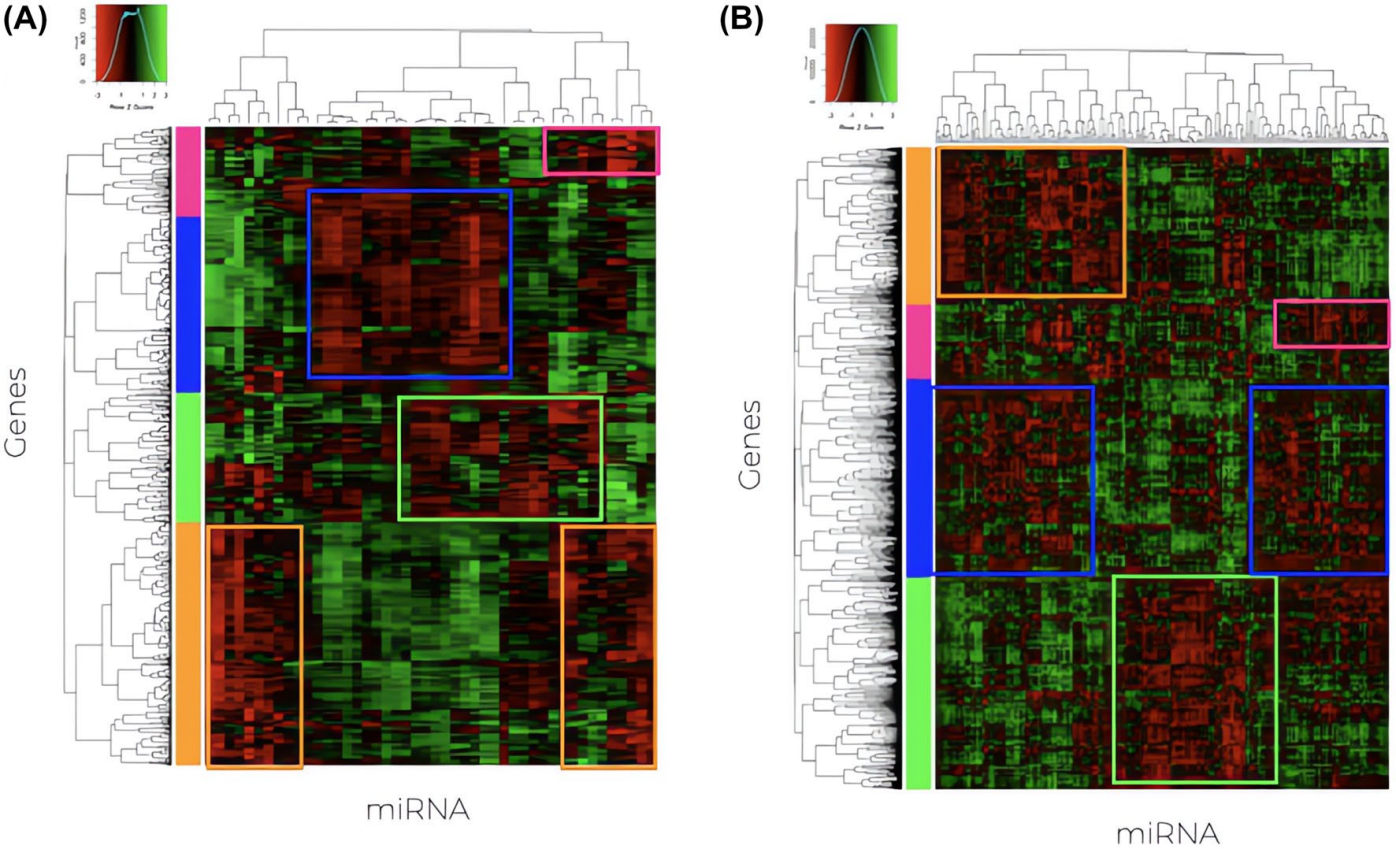
Heatmap of Pearson’s correlation coefficients between miDE and gDE in LUAD (**A**) and lSCC (**B**). The boxes mark the detected groups with negative correlation miDE–gDE pairs

Differential expression analysis of the miRNAs (Supplementary Table S2) allowed the identification of 360 miDE in lSCC, of which 202 were upregulated and 158 miDE downregulated. In LUAD patients, 82 miDE were obtained, of which 44 were upregulated and 38 were downregulated. Figure 2 shows the heatmap after the correlation of FC values of the overexpressed miDEs, using Pearson’s coefficient, with the FC values of the gDEs previously identified for the same patients in the same cancers [14].

It can be noticed (Fig. 1 and Supplementary Table S3) that the 202 up-regulated miDE and 3667 gDE in lSCC, produce 6212 miDE–gDE correlated pairs after correcting for $P < 0.05$. These pairs correspond to 198 miDE and 1576 unique gDE in lSCC. On the other hand, in the LUAD, it is observed that, between the 44 up-regulated miDE and the 2818 gDE, fewer significative correlations occur: 1475 miDE–gDE pairs, corresponding to 44 miDE and 786 unique gDE.

Considering that the correlation can be by chance, it is necessary to validate the existence of a real interaction. This validation was performed consulting the databases *DIANA-TarBase* and *DIANA-microT-CDS*. A total of 299 miDE–gDE pairs were confirmed in lSCC (4.8%), while only 57 pairs were confirmed in LUAD (3.8%). These pairs correspond to 94 miDE and 230 gDE unique in lSCC, and 24 miDE and 53 gDE unique in LUAD.

### Construction of regulation network for miRNA–mRNA

**Fig. 3:**
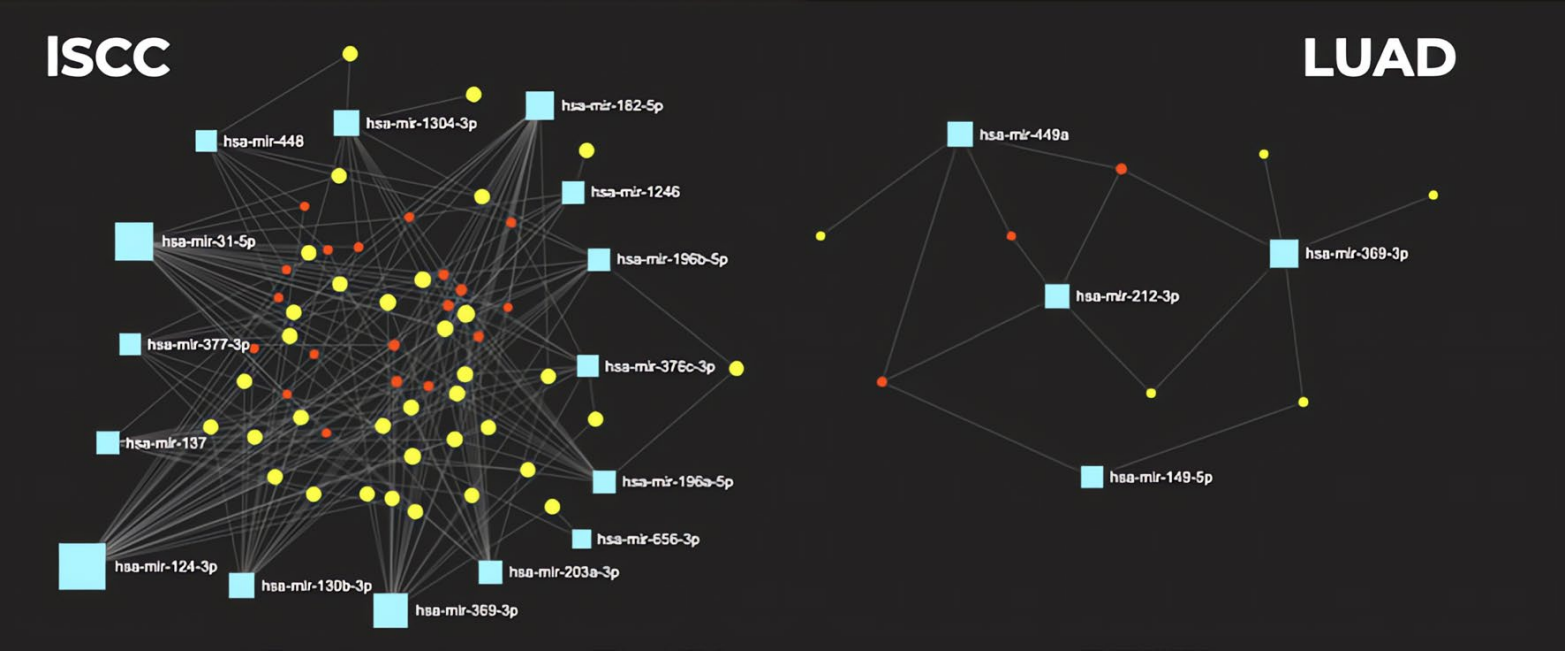
Interaction network with the candidates miDE and gDE built using *miRNet*, in lSCC and LUAD. Candidate miDEs are shown in blue; gDEs with which interaction occurs are in yellow; other mRNAs in intermediate interaction nodes are marked in red

The 94 miDE and 230 gDE unique in lSCC, and the 24 miDE and 53 gDE unique in LUAD were loaded in *miRNet* to build a specific functional interaction network for lSCC and another for LUAD, shown in Fig. 3.

The specific functional interaction network of lSCC consists of 15 candidate miRNAs that regulate the repression of 30 mRNAs. In comparison, the specific functional interaction network of LUAD consists of four candidate miRNAs that seem to handle the repression of five mRNA. All the candidates are shown in the Table 1.

**Table 1:** Table of miRNA–mRNA pairs derived from specific interaction network in LUAD and lSCC

miRNAs	mRNA targets
*LUAD*	
hsa-miR-149-5p	*SLC8A1*
hsa-miR-212-3p	*MICU3*
hsa-miR-449a	*CLIC5*
hsa-miR-369-3p	*BMPR2, RASSF8*
*lSCC*	
hsa-miR-203a-3p	*EPB41L3*
hsa-miR-137	*SLCO4C1*
hsa-miR-182-5p	*ARRDC3*
hsa-miR-124-3p	*AMOTL2, ARRDC4*
hsa-miR-1246	*RDX, RTKN2*
hsa-miR-1304-3p	*HIPK1, NFATC2*
hsa-miR-130b-3p	*HEG1, PTPRG*
hsa-miR-376c-3p	*CD47, LIMCH1*
hsa-miR-377-3p	*NECAB1, TAL1*
hsa-miR-448	*MYO5B, REEP5*
hsa-miR-656-3p	*CD55, HHIP*
hsa-miR-31-5p	*ATOH8, CD55, EMCN*
hsa-miR-196b-5p	*BMPR2, RDX, REEP5, TSPAN12*
hsa-miR-196a-5p	*BMPR2, PRTG, RDX, TMEM170B, TSPAN12*
hsa-miR-369-3p	*ATP8A1, IDI1, LAMP3, NECAB1, PDE4D, PREX2*

Functional analysis in DAVID of Table 1 mRNAs revealed that the mRNA *BMPR2*, *ATOH8* are involved in the cell migration process, while the mRNAs *EMCN*, *TAL1*, *TSPAN12* are involved in angiogenesis (Table 2).

**Table 2:** GO terms, according to DAVID, for the the mRNA that form the interaction network in lSCC and LUAD

GO ID	Term (GO principle)	mRNA
GO:0072577	Endothelial cell apoptotic process	*BMPR2, HIPK1*
GO:0060216	Definitive hematopoiesis	*TAL1, HIPK1*
GO:0061028	Establishment of the endothelial barrier	*PDE4D, RDX*
GO:0051443	Positive regulation of the activity of the protein ubiquitin transferase	*ARRDC4, ARRDC3*
GO:0010842	Formation of the retina layer	*HIPK1, TSPAN12*
GO:0031032	Organization of the actomyosin structure	*LIMCH1, EPB41L3*
GO:0002027	Heart rate regulation	*PDE4D, SLC8A1*
GO:0001525	Angiogenesis	*EMCN, TAL1, TSPAN12*
GO:0030501	Positive regulation of bone mineralization	*BMPR2, SLC8A1*
GO:0010595	Positive regulation of endothelial cell migration	*BMPR2, ATOH8*
GO:0071320	Cellular response to cAMP	*PDE4D, SLC8A1*
GO:0030097	Hematopoiesis	*TAL1, RTKN2*

### Validation of expression and secretion in fluids

**Fig. 4:**
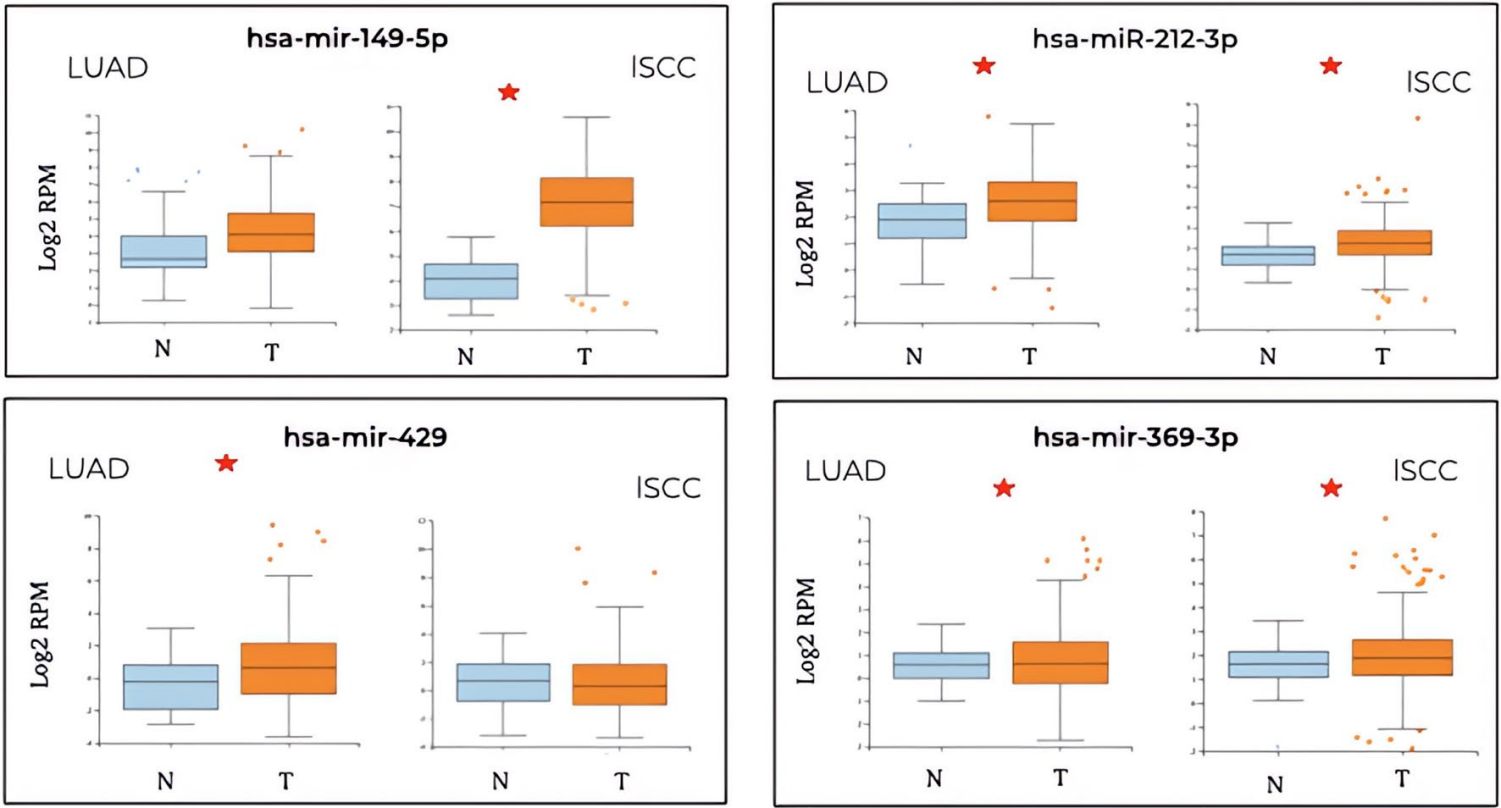
Validation of the differential expression between tumor and healthy tissues of the 4 candidate miDEs in LUAD according to the larger cohort in *MIR-TV*. On the *x*-axis, the expression levels are presented in the form of log2RPM (readings mapped per million on a logarithmic basis). The miDEs with a significant difference in expression between tumor and healthy tissue are shown with an *

**Fig. 5:**
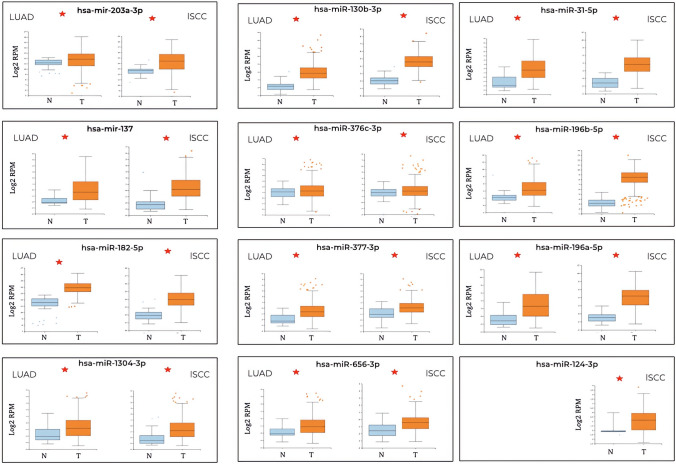
Validation of the differential expression between tumor and healthy tissues of the 15 candidate miDEs in lSCC according to the larger cohort in *MIR-TV*. On the *x*-axis, the expression levels are presented in the form of log2RPM (readings mapped per million on a logarithmic basis). The miDEs with a significant difference in expression between tumor and healthy tissue are shown with an *

Inspection of the differential expression in a larger cohort, accessible from *MIR-TV*, indicated that, for LUAD, the network was formed by four miDE, where the hsa-miR-149-5p did not show a significant expression change between tumor and healthy tissue, so it is discarded from the study (Fig. 4). The candidate hsa-miR-149-5p is only significantly expressed in LUAD, not in lSCC. The candidates hsa-miR-212-3p and hsa-miR-369-3p are significantly expressed in the tumor tissue of both types of cancer (Fig. 4). When the interaction network in lSCC was considered, composed by 15 miDE, hsa-miR-1246 and hsa-miR-448 were discarded since no significant change was observed in *MIR-TV* (Fig. 5). The candidate hsa-miR-124-3p would be specific for the lSCC tumor type in the absence of expression data in LUAD (Fig. 5). The rest of the possible candidates show a significant expression difference in tumor tissue both in lSCC and LUAD (Fig. 5). Hence, the potential candidates for biomarkers are reduced to three miDE in the interaction network for LUAD and 13 miDE for lSCC.

The candidate miRNAs with differential expression confirmed were the investigated in extracellular fluids, determining its potential importance as a biomarker in liquid biopsies. To do so, their expression was inspected in the database of miRNA of extracellular liquids, *liqDB*, and the results are shown in Table 3. In LUAD, hsa-miR-369-3p, miDE common to LUAD and lSCC, would be completely ruled out as it was not detected in any of the extracellular fluids studied. However, Table 3 shows that hsa-miR-212-3p and hsa-miR-449a could be helpful biomarkers in liquid biopsies since they can be detected at expression levels of between 10 and 100 RPM in plasma. Furthermore, all of them are detectable in bronchoalveolar lavages. On the contrary, none of them has been identified in exosomes.

Regarding the lSCC candidates, the hsa-miR-196a-5p, hsa-miR31-5p, hsa-miR-369-3p and hsa-miR-376c-3p are discarded because they do not appear in any extracellular fluid inspected; despite this, they could be used in tissue biopsies as they have been detected in bronchoalveolar lavages. Therefore, of the 13 possible biomarkers only 9, hsa-miR124-3p, hsa-miR-1304-3p, hsa-miR-130b-3p, hsa-miR-137, hsa-miR-182-5p, hsa-miR-196b-5p, hsa-miR-203a-3p, hsa-miR-377-3p, and hsa-miR-656-3p, are detectable in plasma, serum, or exosomes.

**Table 3:** Expression level of candidate miDEs in plasma, serum, exosomes and bronchoalveolar lavage

miRNA	Plasma	Serum	Exosomes	Bronch.
*LUAD*				
hsa-miR-369-3p	−	−	−	+
hsa-miR-212-3p	+	−	−	+
hsa-miR-449a	+	−	−	+
*lSCC*				
hsa-miR-196a-5p	−	−	−	−
hsa-miR-31-5p	−	−	−	+
hsa-miR-369-3p	−	−	−	+
hsa-miR-376c-3p	−	−	−	−
hsa-miR-124-3p	+	−	−	+
hsa-miR-1304-3p	−	+	−	−
hsa-miR-137	+	−	−	−
hsa-miR-196b-5p	−	+	−	+
hsa-miR-377-3p	++	−	−	−
hsa-miR-656-3p	++	−	−	−
hsa-miR-130b-3p	+	+	+	+
hsa-miR-182-5p	+	+++	+++	+++
hsa-miR-203a-3p	+	+	++	+++

‘+’ indicates expression values between 10–100 RPM,‘++’ indicates expression values between 101 and 1000 RPM and ‘+++’ indicates expression values greater than 1001 RPM. Bronch.: bronchoalveolar lavage

**Fig. 6:**
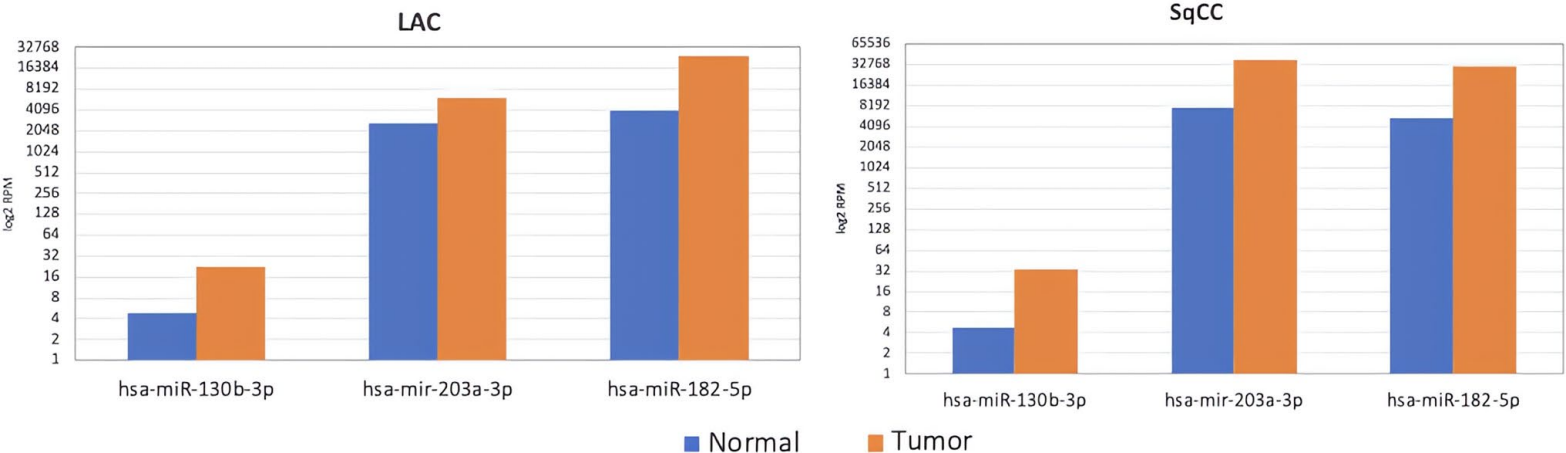
Quantification of miRNA content from exosomes secreted from healthy and tumoral tissue from lung cancer patients represented as logarithm of RPM

Investigating the *ERV DB* database, it can be suggested that hsa-miR-130b-3p, hsa-miR-182-5p and hsa-miR-203a-3p, detected in exosomes, could come from vesicles secreted by tumor lung tissue, since their level is higher in exosomes from tumor tissue than from healthy tissue, both in lSCC and LUAD (Fig. 6). This knowledge would open a new field of study that could monitor the evolution of the disease as the tumor tissue directly secretes them.

All of the above leads us to establish a possible gene signature composed of 11 miDE summarized in Table 4, of which the hsa-miR-449a would be a possible LUAD-specific circulating miRNA biomarker and hsa-miR-124-3p would be specific to lSCC. The remaining nine possible biomarkers do not appear to differentiate between lSCC or LUAD. We want to highlight that hsa-miR-130b-3p, hsa-miR-182-5p and hsa-miR-203a-3p are specific for exosomes secreted by tumor tissue (Table 4).

**Table 4:** Final proposed biomarkers for liquid biopsies

miRNA	Tumor	c_biomarker	e_biomarker	l_biomarker
hsa-miR-212-3p	LUAD/lSCC	+	−	+
hsa-miR-449a	LUAD	+	−	+
hsa-miR-124-3p	lSCC	+	−	+
hsa-miR-1304-3p	LUAD/lSCC	+	−	−
hsa-miR-137	LUAD/lSCC	+	−	−
hsa-miR-196b-5p	LUAD/lSCC	+	−	+
hsa-miR-377-3p	LUAD/lSCC	+	−	−
hsa-miR-656-3p	LUAD/lSCC	+	−	−
hsa-miR-130b-3p	LUAD/lSCC	+	+	+
hsa-miR-182-5p	LUAD/lSCC	+	+	+
hsa-miR-203a-3p	LUAD/lSCC	+	+	+

* c_biomarker* detectable circulating miRNA in plasma or serum;* e_biomarker* miRNA detectable in exosomes;* l_biomarker* detectable miRNA in bronchoalveolar lavage

## Discussion

The present work is based on the differential expression changes between miRNA and mRNA in lSCC and LUAD. From the point of view of gene dysregulation, the total number of gDEs in lSCC (3667) is 23.3% higher than in LUAD (2818). This same trend is maintained when we observe the total number of miDEs, where the percentage in lSCC (360) is 77.3% higher than in LUAD (202). These profiles could be indicating a higher level of gene dysregulation in lSCC compared to LUAD, which could be caused by external factors such as tobacco, is in agreement with the evidence that shows that LUAD is the most frequent lung cancer among the non-smokers, and therefore less exposed to carcinogenic substances [27, 28]. On the other hand, miDE–gDE correlation, as well as gene interaction studies, verify that 15 miDE–gDEs pairs in lSCC and 4 pairs in LUAD interact significantly with each other, forming a specific interaction network for lSCC and another for LUAD. Thus, these interactions correspond to 19 unique miRNAs that repress 35 unique mRNAs, of which some of them are related to angiogenesis and cell migration (Tables 1 and 2).

Validation of the differential expression in larger cohorts ruled out only hsa-miR-149-5p, hsa-miR-1246, hsa-miR-448, which showed that the integration of mRNA and miRNA expression data generates very robust results, even from very small cohorts, such as those used in this work.

The expression in tumor tissue of hsa-miR-369-3p, common to both types of cancer, seems to be involved in different functional processes depending on the tumor type. In LUAD this miRNA seems down-regulated the expression of the mRNAs (*BMPR2*, *RASSF8*), meanwhile, in lSCC seems down-regulate the expression of the mRNAs (*ATP8A1*, *IDI1*, *LAMP3*, *NECAB1*, *PDE4D*, *PREX2*). However, the lack of detection in extracellular fluids of hsa-miR-369-3p, together with hsa-miR-196a-5p, hsa-miR31-5p and hsa-miR-376c-3p has led us to discard them as possible non-invasive biomarkers, although they could be candidates in tumor tissue biopsies.

The hsa-miR-449a, hsa-miR-212-3p, hsa-miR-124-3p, hsa-miR-1304-3p, hsa-miR-137, hsa-miR-196b-5p, hsa-miR-377-3p and hsa-miR-656-3p are candidate circulating miRNA detection biomarkers for LUAD and lSCC. Among them, hsa-miR-449a could be specific for LUAD as it is not significantly expressed in tissue in lSCC, while hsa-miR-124-3p would be specific for lSCC as it is not described as miDE in LUAD. The candidates hsa-miR-377-3p and hsa-miR-656-3p present high levels of detection in plasma, but not in bronchoalveolar lavage, suggests differential secretion by cell type in tumor development.

Possible biomarkers hsa-miR-203-3p, hsa-miR-130b-3p and hsa-miR-182-5p show special interest when they are specifically identified in exosomes secreted by tumor tissue, both in LUAD and in the lSCC (Fig. 6).

Analyzing their relationship as possible biomarkers secreted in exosomes by tumor tissue, hsa-miR-203a-3p has been described as negatively regulated in both gastric cancer and gastric cancer cell lines, showing that its overexpression reduces the proliferation of cancer cells [29]. However, our results show the opposite behavior, as it is strongly detected in exosomes secreted by the tumor tissue. Furthermore, this same miRNA has already been proposed as a biomarker for the detection of lung cancer from plasma using qPCR [30].

In bladder cancer, it has been shown that hsa-miR-130b-3p could inhibit the expression of the *PTEN* gene, that which promotes proliferation, migration, invasion and rearrangement of the cytoskeleton by activating the pathway signaling *PI3K*; these same authors propose that the inhibition of this miRNA could induce cell apostosis [31]. Likewise, this miRNA has already been described as an NSCLC oncogene [32]. These evidences corroborate the importance of hsa-miR-130b-3p as a possible diagnostic biomarker specify in lung cancer.

The hsa-miR-182-5p should be highlighted due to its high level of detection in bronchoalveolar lavage, serum, and exosomes. Furthermore, accumulating evidence indicates that dysregulation of hsa-miR-182-5p can serve as diagnostic and pronostic biomarkers for some cancers. The literature reported the functionality of hsa-miR-182-5p in different types of cancer, such as renal cell carcinoma and liver cancer [33, 34], although it is not yet clear in lung cancer. In this sense, a recent study [35] shows that this miRNA is overexpressed in tumor tissue and in the peripheral blood of patients with NSCLC; in addition, and according to the same authors, its inhibition suppresses cell proliferation and increases apoptosis in NSCLC cell culture.

Last, we cannot consider the miDE detectable only in bronchoalveolar lavage as non-invasive biomarkers, since the technique itself is invasive.

In conclusion, a miDE–gDE interaction network specific for lSCC and another specific for LUAD are presented, which work as active regulators of gene expression. These interaction network allowed the identification of 11 candidate miRNAs for biomarkers in non-invasive lung cancer biopsy (Table 4), of which hsa-miR-449a would be specific for LUAD, while hsa-miR-124-3p would be specific to lSCC. On the other hand, the possible candidates hsa-miR-130b-3p, hsa-miR-203a-3p and hsa-miR-182-5p have a high detection rate in exosomes secreted by lung tissue, and seem to have a clear relationship with tumor development, so its in-depth study could shed light on the processes of tumor growth and development. Following these results, we propose that *in silico* studies, based on mRNA and miRNA expression data, may constitute a useful tool in the identification of new non-invasive biomarkers that help in the early diagnosis and prognosis of cancer lung.

## Supplementary Information

Below is the link to the electronic supplementary material.

Supplementary material: Three supplementary tables are complementing the manuscript: **Supplementary Table S1:** Supplementary Table S1: Summary of the raw data sequencing generated for each of the samples.

Supplementary Table S2: Summary of the miDEs in LUAD and lSCC described in this work. Each sheet contains gene ID, $logFC_{OE}$, statistical test parameter, \textit{P}-value and FDR of every miDEs in each one of the lung cancers studied in this work.

Supplementary Table S3: Summary of the Pearson{\rsquo}s correlation coefficients.

## Data Availability

LUAD and lSCC sequencing reads are available at Bioproject PRJNA727087.

## Code availability

All scripting code can be obtained upon request to Elena Espinosa García (emesga@uma.es).
